# Skeletal Muscle Microvascular Dysfunction in Obesity-Related Insulin Resistance: Pathophysiological Mechanisms and Therapeutic Perspectives

**DOI:** 10.3390/ijms23020847

**Published:** 2022-01-13

**Authors:** Chiedozie Kenneth Ugwoke, Erika Cvetko, Nejc Umek

**Affiliations:** Institute of Anatomy, Faculty of Medicine, University of Ljubljana, 1000 Ljubljana, Slovenia; chiedozie-kenneth.ugwoke@mf.uni-lj.si (C.K.U.); erika.cvetko@mf.uni-lj.si (E.C.)

**Keywords:** obesity, microvascular dysfunction, insulin resistance, skeletal muscle, obesity treatment

## Abstract

Obesity is a worrisomely escalating public health problem globally and one of the leading causes of morbidity and mortality from noncommunicable disease. The epidemiological link between obesity and a broad spectrum of cardiometabolic disorders has been well documented; however, the underlying pathophysiological mechanisms are only partially understood, and effective treatment options remain scarce. Given its critical role in glucose metabolism, skeletal muscle has increasingly become a focus of attention in understanding the mechanisms of impaired insulin function in obesity and the associated metabolic sequelae. We examined the current evidence on the relationship between microvascular dysfunction and insulin resistance in obesity. A growing body of evidence suggest an intimate and reciprocal relationship between skeletal muscle microvascular and glucometabolic physiology. The obesity phenotype is characterized by structural and functional changes in the skeletal muscle microcirculation which contribute to insulin dysfunction and disturbed glucose homeostasis. Several interconnected etiologic molecular mechanisms have been suggested, including endothelial dysfunction by several factors, extracellular matrix remodelling, and induction of oxidative stress and the immunoinflammatory phenotype. We further correlated currently available pharmacological agents that have deductive therapeutic relevance to the explored pathophysiological mechanisms, highlighting a potential clinical perspective in obesity treatment.

## 1. Introduction

Obesity (body mass index (BMI) ≥ 30.0 kg/m^2^) [1] is an escalating global health challenge affecting 13% of the world’s population, according to recent World Health Organization estimates [2]. Over the past five decades, the global prevalence of obesity has risen to pandemic proportions [3,4]. The rising global trend of obesity is associated with the increasing prevalence of diabetes mellitus (DM) type 2, hypertension and other cardiovascular morbidities, liver disease, and malignancies [5,6]. Much of the obesity-related mortality is due to cardiovascular disease, but although the epidemiological links between obesity and a broad spectrum of cardiometabolic disorders are clearly recognised, the specific pathophysiological mechanisms are not yet fully understood [6,7].

Skeletal muscle accounts for 40–50% of the total body mass and structurally consists of multiple fascicles or bundles of different physiochemically and metabolically distinct fibre types, classified based on the expression of different myosin heavy-chain isoforms [8,9]. The skeletal muscle represents the largest endocrine tissue involved in glucose metabolism, mediating ~80% of insulin-stimulated glucose uptake under euglycemic hyperinsulinaemic conditions [10]. Decreased sensitivity for insulin-mediated glucose uptake in skeletal muscle is a core pathophysiological denominator in obesity-related alterations in metabolic phenotype [11]. However, the exact mechanisms of such attenuated biological response are not fully understood. Skeletal muscle microvascular and metabolic physiology and pathophysiology are closely linked, and a growing body of evidence has confirmed the critical role of microvascular dysfunction (inadequate microvascular response to physiologic metabolic demand or challenge) in the mediation of obesity-related insulin resistance [12,13,14,15]. Several pathophysiological mechanisms in obesity, e.g., oxidative stress, alterations in adipokine secretion, decreased adiponectin levels, increased inflammatory mediators, and increased activation of the renin–angiotensin system, may contribute to impaired microvascular dilatation and insulin-mediated capillary recruitment, leading to suboptimal glucose and insulin delivery to the skeletal muscle, and subsequent impaired glucose homeostasis [16,17,18,19,20]. Previously, microvascular dysfunction was merely regarded as a diabetic sequela, manifesting as classical microangiopathic complications such as retinopathy, nephropathy, and neuropathy. Current evidence, however, demonstrates that microvascular dysfunction and hyperglycaemia exhibit a bidirectional relationship: microvascular dysfunction antedates and mediates hyperglycaemia in insulin-resistant states, while being a known consequence of prediabetic and diabetic levels of hyperglycaemia [21,22]. It has equally been shown that microvascular and macrovascular complications share similar risk factors and reciprocal pathophysiological mechanisms [23].

In chronic obesity, microvascular dysfunction has been shown to mediate impaired insulin sensitivity and β-cell dysfunction via multifactorial mechanisms, providing scaffolding for subsequent hyperglycaemic sequelae and complications, including multiorgan microangiopathy [13,21,24,25]. Therefore, targeting microvascular dysfunction in this vicious cycle may provide an important pharmacotherapeutic window for preventing or abrogating obesity-related insulin resistance and its ramifications. Recent clinical evidence shows that optimizing glycaemic control improved microvascular function in early but not advanced phase of DM type 2, suggesting a strong benefit of initiating early aggressive interventions to prevent or attenuate the progression of microvascular complications and insulin-resistant phenotypes [26]. The aim of this paper was to review the general background of the relationship between skeletal muscle microvascular and metabolic physiology, as a point of departure to explore the etiological role of microvascular dysfunction in obesity-related insulin resistance. Furthermore, it also highlights the therapeutic implications of the elucidated pathophysiological mechanisms and correlates the currently available or potential pharmacological agents that bear important therapeutic relevance.

## 2. Vascular and Metabolic Physiology of Skeletal Muscle Microcirculation

### 2.1. Anatomical Background

Microcirculation includes all vessels less than 150 μm in diameter, namely capillaries, venules, and third- or fourth-order arterioles [27]. However, this definition excludes larger arterioles with important microcirculatory function. A more inclusive definition based on vessel physiology considers all vessels that myogenically alter the luminal diameter in response to increased pressure as part of the microvasculature [14,27]. Arterioles consist of a layer of smooth muscle cells surrounding a layer of endothelial cells, while capillaries consist of a monolayer of squamous endothelial cells without a muscle layer. The microcirculation represents much of the total vascular surface area and controls the delivery of oxygen and nutrients to tissues by regulating capillary vascular resistance and trans-endothelial exchange of blood solutes.

Although the skeletal muscle’s microvascular histological architecture varies according to muscle typology and location, the basic gross anatomical characteristics are shared. One or more feed arteries in the epimysium ramify into an intertwined network of arterioles in the perimysium, which then branch at regular intervals into transverse arterioles that pierce the endomysium and asymmetrically divide into terminal arterioles that give rise to capillary networks running parallel to the muscle fibres. The venules arise from the contralateral loop of the capillary arcade and ramify progressively into larger venules in tandem with the arteriolar branching. Each muscle fibre is perfused by multiple terminal arterioles and capillary units along its length, and the loop of capillaries supplied by one terminal arteriole and drained by a venule constitutes the microvascular unit which represents the basic functional unit of skeletal muscle microcirculation [28].

### 2.2. The Microvascular Endothelium Is the Key Regulator of Vascular Homeostasis

The regulation of microvascular function is controlled by the balance in the activation of the sympathetic and parasympathetic nervous system, as well as by local metabolic and myogenic autoregulatory mechanisms. The microvascular endothelium plays multifaceted biological functions, including serving as a semipermeable physiological barrier and component of innate immunity, as well as regulation of vascular tone, mechano-transduction, procoagulant/anticoagulant balance, endothelial repair, and angiogenesis, among other functions [29]. It produces both vasodilators/anti-thrombogenic factors and vasoconstrictors/prothrombotic factors. The former include nitric oxide (NO), prostacyclin, bradykinin, and endothelium-derived hyperpolarizing factor, while the latter consists of endothelin-1 (ET-1), angiotensin II (Ang II), thromboxane A2 (TXA2), prostacyclin H2, and superoxide [30]. These vasoactive molecules may act in autocrine, paracrine, or endocrine fashion to regulate vessel tone and diameter, proliferation of vascular smooth muscle cells (VSMC), and activation and adhesion of platelets and leukocytes.

NO is the most characterized endothelium-derived relaxing factor and is synthesized by the calcium-calmodulin-dependent endothelial nitric oxide synthase (eNOS) from L-arginine substrate, with tetrahydrobiopterin (BH_4_) as co-factor. Various mechanisms of eNOS activation have been identified, including phosphorylation, glutathionylation, *S*-nitrosylation, and *N*-acetyl glycosylation [31]. Insulin induces insulin receptor substrates (IRS) 1 and 2 signalling, which activates the phosphatidylinositol 3-kinase (PI3K) and protein kinase B (Akt), to phosphorylate eNOS at Ser1177 (in humans), catalysing the conversion of L-arginine to L-citrulline and NO [32]. Several cofactors, including calmodulin, flavinmononucleotide, flavin adenine dinucleotide, and nicotinamide adenine dinucleotide phosphate, are involved in this reaction [33]. NO diffuses to the vascular smooth muscle cells, where it mediates vasodilatation by activating guanylate cyclase to catalyse increased cyclic guanosine monophosphate (cGMP) synthesis [34].

### 2.3. Role of Insulin in the Regulation of Microvascular Tone

Vascular smooth muscle tone is maintained by the dynamic balance of the endothelium-derived relaxing and contracting factors. This vasomotor balance may shift in response to both mechanical factors such as enhanced shear stress, and endocrine factors such as insulin. Insulin is a potent hormone produced by the pancreatic β-cell of the Islets of Langerhans, which exerts a wide range of anabolic effects, including promoting glucose uptake in skeletal muscles and adipocytes, glycogen synthesis in skeletal muscles, and triacylglycerol synthesis in adipocytes and suppressing glucose production in hepatocytes and lipolysis in adipocytes [35].

In healthy vasculature, insulin predominantly mediates microvascular dilatation by activating PI3K with consequent eNOS activation and NO production, although it can also mediate vasoconstriction by increasing ET-1 and vasoconstrictor eicosanoids via the intracellular mitogen-activated protein kinase (MAPK) signalling pathway and the extracellular signal-regulated kinase-1/2 (ERK-1/2) pathway [30,36,37]. Insulin signal transduction via the PI3K/Akt pathway also modulates vascular immuno-inflammation by increasing vascular endothelial growth factor (VEGF) and hemeoxygenase-1 expression and decreasing vascular cell adhesion molecule 1(VCAM-1) expression [38].

The ET-1/NO balance is maintained in favour of insulin-stimulated vasodilatation, which enhances downstream capillary perfusion and trans-endothelial transport of insulin. Direct visualisation of the trans-endothelial transport of fluorescent insulin shows that the movement to the skeletal muscle interstitium occurs by a fluid-phase transport mechanism that is receptor-dependent and regulated by the balance of oncotic and hydrostatic pressures [39]. The ability of insulin to dose- and time-dependently increase total skeletal muscle blood flow and consequent insulin-mediated glucose uptake via dilatation of resistance vessels was first reported by Baron and colleagues about three decades ago and demonstrates the link between the vascular and metabolic function of insulin [40,41,42].

### 2.4. Functional Capillary Recruitment

Furthermore, it has also been established that without increasing total blood flow, insulin can selectively redirect microvascular circulation in favour of perfusion of nutritive capillary beds by decreasing precapillary arteriolar tone or altering arteriolar vasomotor response, facilitating glucose delivery and uptake in skeletal muscle [43,44]. During physiologic hyperinsulinaemia or glucose challenge, contrast-enhanced ultrasound has been used to demonstrate in vivo that capillary recruitment is an early forerunner phenomenon to muscle glucose uptake, ensuring a maximal metabolic effect of insulin [45]. The resistance arterioles that regulate total blood flux were shown to be less insulin-sensitive than the precapillary arterioles mediating microvascular recruitment [44]. This functional capillary recruitment which accounts for about 40% of insulin-mediated muscle glucose uptake is dependent on the activation of endothelial PI3K pathway including autophosphorylation of the insulin receptor, phosphorylation of tyrosine residues of the IRS-1 and 2, and phosphorylation of phosphoinositide-dependent kinase 1 (PDK-1) and Akt, leading to the translocation of glucose transporter-4 (GLUT-4) to the cell membrane, which is the rate-limiting process for skeletal muscles’ insulin-mediated glucose uptake [14,15,46].

### 2.5. Assessment of Skeletal Muscle Microvascular Structure and Function

Microvasculature research remains relatively underdeveloped, largely on account the limitations in techniques for morphological and functional studies. Accordingly, it is hoped that our understanding of the physiological mechanisms of microvascular function will continue to evolve as new study techniques emerge. Currently, microvascular function in skeletal muscles can be measured by plethysmography, contrast-enhanced ultrasonography, intravital microscopy, plasma concentration of several endothelial biomarkers, and other surrogate clinical markers such as urinary albumin excretion [36,44,45,47,48,49,50,51]. Additionally, application of various stimuli including local ischaemia, temperature changes, and vasoactive agents such as acetylcholine, adenosine, serotonin, bradykinin, and sodium nitroprusside, can be used to study microvascular response. Histological assessment of skeletal muscle microvasculature is conventionally accomplished by two-dimensional (2D) analyses of tissue cross-sections [52,53], although recently, a three-dimensional (3D) analytic technique that overcomes the usual technical biases and inconsistencies associated with the traditional 2D approach has been proposed [54,55,56].

## 3. Skeletal Muscle Microvascular Dysfunction in Obesity

**Figure 1:**
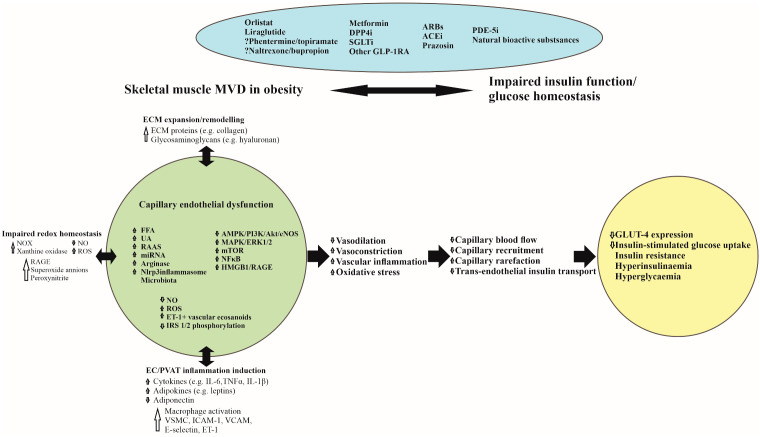
Pathophysiological mechanisms linking skeletal muscle microvascular dysfunction with glucometabolic disorder in obesity and potential therapeutic agents. ACEi, Angiotensin-Converting Enzyme inhibitors; AMPK 5’-, Adenosine Monophosphate-activated Protein Kinase; ARBs, Angiotensin Receptor Blockers; Akt, Protein Kinase B; DPP4i, Dipeptidyl-Peptidase-4 inhibitors; EC, Endothelial Cell; ECM, Extracellular Matrix; eNOS, endothelial Nitric Oxide Synthase; ERK, Extracellular-signal-regulated Kinase; ET-1, Endothelin 1; FFA, Free Fatty Acids; GLP-1RA, Glucagon-like Peptide-1 Receptor Agonists; GLUT-4, Glucose Transporter 4; HMGB 1, High Mobility Group Box chromosomal protein 1; IRS, Insulin Receptor Substrate; ICAM1, Intercellular Adhesion Molecule 1; IL-1β, Interleukin-1Beta; IL-6, Interleukin-6; MAPK, Mitogen-Activated Protein Kinase; miRNAs, Micro RNAs; mTOR, Mammalian Target of Rapamycin; MVD, Microvascular Dysfunction; NF-κB, Nuclear Factor-Kappa B; *NO*, Nitric Oxide; *NOX*, Nicotinamide Adenine Dinucleotide Phosphate (NADPH) Oxidase; PDE-5i, Phosphodiesterase-5 inhibitors; PI3K, Phosphatidylinositol 3 Kinase; RAAS, Renin-Angiotensin-Aldosterone System; ROS, Reactive Oxygen Species; RAGE, Receptor for Advanced Glycation End products; SGLT2i, Sodium Glucose co-Transporter 2 inhibitors; TNF-α, Tumour Necrosis Factor-alpha; UA, Uric Acid; VCAM1, Vascular Cell Adhesion Molecule 1; VSMC, Vascular Smooth Muscle Cells.

Obesity is associated with a broad spectrum of metabolic derangements including hyperglycaemia, insulin resistance, and a proinflammatory milieu, all of which contribute to vascular endothelial vasodilator and fibrinolytic dysfunction and extracellular matrix remodelling [47]. Insulin resistance precedes the development of hyperglycaemia and DM type 2 and results in compensatory hyperinsulinaemia, which contributes to increased inflammation and oxidative stress. Elevated plasma free fatty acids (FFAs) is considered an important etiologic factor linking insulin resistance, oxidative stress, and inflammation with obesity and other cardiometabolic disorders, and impaired insulin-mediated glucose uptake correlates with circulating FFA levels [57,58]. The increased circulating free fatty acids in obesity probably triggers the early phase of microvascular dysfunction via downregulation of the of the endothelial AMPK-PI3K-Akt-eNOS pathway [59], while other factors such as alterations in adipokines (e.g., leptins, adiponectin, monocyte chemotactic protein-1, and retinol binding protein 4) and inflammatory cytokines (e.g., interleukin-6 (IL-6), and tumour necrosis factor-alpha (TNFα)) released from both visceral and perivascular adipose tissue, help to drive the progression of the dysfunction (Figure 1) [60,61,62]. Besides visceral or extracellular lipids, accumulation of saturated lipid droplets, mainly triglycerides, in skeletal muscle fibres has been shown to have a pathogenic role in insulin resistance [63]. It was recently demonstrated that such intramyocellular lipid accumulation exhibit both muscle- and fibre-type specificity in obese mice, meaning that similar muscle fibres in different muscles may show different pattern of lipid accumulation [9]. Moreover, it was also shown that capillary network changes in obesity are muscle-fibre-specific, being more pronounced around small and more oxidative muscle fibres than around large fibres [56].

### 3.1. Skeletal Muscle Microvascular Functional and Structural Dynamics in Obesity

Skeletal muscle microvascular perfusion, blood flow dynamics, and insulin permeability are critical determinants of insulin action in skeletal muscles and have become a compelling focus of investigation in studying disorders of glucose metabolism. While some studies have described relatively preserved skeletal muscle blood flow in obese young adult humans [64,65,66], several other investigations in obese humans and animal models suggest a blunted vascular conductance that is independent of age and vascular bed [24,67,68,69,70,71]. In a systematic review and meta-analysis probing the association between BMI and retinal vascular calibre, a surrogate marker of microvascular disease, Boillot et al. noted a narrower retinal arteriolar and wider venular calibres in both adults and children with increasing BMI, affirming that the biological mechanisms of microvascular dysfunction are similar across organs and independent of age [16].

Obesity is associated with decreased endothelial NO production, decreased insulin-stimulated vasomotion, and reduced capillary density, leading to impaired insulin-mediated capillary recruitment and microvascular dilatation [24]. During an insulin clamp, impaired capillary insulin delivery in humans with prediabetes and mouse models of insulin resistance results in increased insulin concentration gradient from plasma to the interstitial fluid [51,72,73]. Skeletal muscle perfusion is determined by changes in the flux rate through individual capillaries and the number of actively perfused capillaries. The capacity of vasodilators such as the phosphodiesterase (PDE5) inhibitor sildenafil to improve vascular function and prevent diet-induced insulin resistance in obese mice [74,75] provides indirect evidence that impaired capillary blood flow is an important mechanism of the development of obesity-related insulin resistance and the progression of prediabetes to diabetes. Conversely, Chadderdon et al. noted that in the early phase of high-fat diet-induced obesity in *rhesus macaques*, an increased basal and glucose-mediated capillary blood volume via endothelial-derived vasodilator pathways, may represent a compensatory mechanism for insulin resistance [76].

Besides changes in microvascular blood flow dynamics, structural changes in capillaries may contribute to insulin resistance [56]. In obesity, actual reduction of microvessel per given tissue volume, or structural capillary regression, has been suggested to be biphasic: an early phase characterized by increased leukocyte adhesion/rolling, oxidant stress, tumour necrosis factor-alpha (TNF-α) levels, and vascular TXA2 and a later phase mediated by impaired NO-bioavailability [71,77]. Several mechanisms including endothelial dysfunction, oxidative stress, apoptosis, and other antiangiogenic factors are thought to underlie capillary regression. Capillary rarefaction in skeletal muscle vascular endothelial growth factor (VEGF)-knockout mice was associated with reduced skeletal muscle insulin-stimulated glucose uptake and glucose tolerance [78]. In contrast to the suggestions that capillary regression contributes to the pathogenesis of skeletal muscle insulin resistance, increased skeletal muscle capillarisation has been demonstrated both in a mouse model of early-stage obesity with insulin resistance [56,79] and in middle-aged men with impaired glucose tolerance precedent to the development of DM type 2 [80]. Thus, the precise role of skeletal muscle capillary rarefaction in the pathophysiology of insulin resistance remains a subject for continued investigation.

Remarkably, sexual dimorphism in skeletal muscle functional and metabolic properties have been well-described in animal models in terms of fibre typology, microvascular architecture, and transcriptomics profile, reflecting the genomic and non-genomic influences of reproductive hormones and sex-specific gene expression [81,82]. In obesity, sex differences have been reported in the interplay of skeletal muscle microvascular dysfunction and metabolic dysregulation. High-fat-diet-induced obesity in male C57BL/6 mice was associated with impaired vasoconstriction in second-order arterioles compared to male control, whereas diet-induced obesity in the female model resulted in significant alterations in both arteriolar vasodilation and vasoconstrictor responses compared to a female control [83].

### 3.2. Endothelial Dysfunction Is the Key Driver of Microvascular Dysfunction in Obesity

As already noted, the vascular endothelium plays a critical role in the regulation of vascular permeability and tone, and endothelial vasodilator dysfunction in the peripheral microcirculation is a hallmark of chronic obesity and insulin resistance and precedes the development of DM type 2 [18,24,68,69,70]. Using peripheral arterial tonometry and laser Doppler flowmetry, van der Heijden et al. recently demonstrated that higher BMI was significantly associated with impaired endothelial function even after adjustment for confounding risk factors such as diabetes mellitus, hypertension, hypercholesterolemia, and smoking [18]. Endothelial cell dysfunction results in impaired NO bioavailability, and enhanced platelet activation, smooth muscle cell proliferation, and adhesion molecule expression [17,84,85,86,87]. In addition, obesity is associated with ultrastructural alterations in the skeletal muscle capillary endothelium, which impair trans-endothelial insulin transport, a critical step in skeletal muscle glucose uptake [51].

Insulin-stimulated NO-dependent skeletal muscle microvascular dilatation involves several mechanisms including activation of the insulin receptor, IRS1 and 2, and the PI3K-Akt-eNOS pathway [32,88]. Decreased expression of IRS-1 and 2 and decreased phosphorylation of Akt and eNOS are key hallmarks of vascular insulin resistance [37,48]. Attenuation of insulin-induced capillary recruitment and consequent reduction in glucose uptake by skeletal muscle was demonstrated in tissue-specific knockout mice lacking endothelial IRS-2 [73]. In obesity, insulin-mediated vasoconstriction via the activation of the ERK1/2 pathway becomes dominant, as its activation of the PI3K pathway in endothelial cells is selectively inhibited, consequently blocking downstream capillary recruitment [36,61].

Increased circulating free fatty acids seen in obesity mediates endothelial dysfunction through several mechanisms including decreased tyrosine phosphorylation of IRS-1/2, impaired phosphorylation of eNOS via inhibition of the PI3K/Akt pathway, impaired ATP-induced mobilization, and influx of calcium in endothelial cells, increased ROS production via protein kinase C (PKC)-dependent activation of NADPH oxidase and consequent stimulation of inflammation via NF-κB activation [57,58,62]. Additionally, the role of inflammasome activation in free-fatty-acid-induced endothelial injury in obesity has been increasingly demonstrated [89,90,91]. It was shown that palmitate markedly induces Nlrp3 inflammasome complex formation in microvascular endothelial cells, leading to downregulation of inter-endothelial tight junction proteins ZO-1/ZO-2, which correlates with increased paracellular endothelial permeability [91]. Additionally, chronic exposure to palmitate has been shown to impair autophagic turnover by decreasing lysosomal acidification via suppressed mitochondrial bioenergetics and cellular ATP levels [92]. Autophagy plays a critical role in the maintenance of endothelial NO bioavailability and regulation of oxido-inflammatory balance, and defective autophagic flux contributes to endothelial dysfunction [93].

Obesity and other insulin-resistant phenotypes are associated with hyperuricaemia [94,95,96]. High uric acid concentration has been suggested to induce endothelial dysfunction via the interaction of high-mobility group box chromosomal protein 1 with the receptor for advanced glycation end products (HMGB1/RAGE pathway). In human umbilical vein, uric acid was shown to inhibit eNOS expression and NO production by increasing the intracellular expression and extracellular secretion of HMGB1, enhancing the expression of RAGE, activating NF-κB, and upregulating the levels of adhesion molecules and inflammatory cytokines including ICAM-1, VCAM-1, TNF-α, and IL-6 [97].

Recent studies have indicated that the upregulation of arginase, a dual isoform manganese metalloenzyme of the urea cycle, represents another important mechanism of endothelial dysfunction. Arginase hydrolyses L-arginine to urea and L-ornithine, and because L-arginine is a common substrate of eNOS and arginase, increased expression or activity of the latter reduces eNOS-dependent NO synthesis in the vascular endothelium via substrate competition [98,99,100]. An enhanced vascular activity and expression of arginase has been demonstrated in obesity [101,102,103]; however, the contribution of this to microvascular endothelial dysfunction is attenuated by aging due to the overriding modulation by the high levels of vascular reactive oxygen species (ROS) from age-dependent increased activity of nicotinamide adenine dinucleotide phosphate (NADPH) oxidase [103].

The enhanced activation of the renin–angiotensin–aldosterone system in obesity further contributes to vascular insulin resistance and endothelial dysfunction. Both Ang II and aldosterone induce degradation of IRS-1; the former via the proto-oncogene tyrosine-protein kinase Src, and the latter via a mineralocorticoid receptor-, ROS-, and Src-dependent mechanism [104,105]. Aldosterone promotes insulin resistance via increased insulin-like growth factor (IGF)-1 receptor expression and hybridization with IRS-1, in addition to mediating Ang II-stimulated ERK1/2 phosphorylation in vascular smooth muscle cells [106,107]. Furthermore, more recent evidence suggests that increased expression of Ang II and the activation of mineralocorticoid receptor by aldosterone may activate the mammalian target of rapamycin (mTOR)–S6K1 signal transduction pathway and promote insulin resistance by inducing phosphorylation of serine residues of IRS [108].

Vascular endothelial dysfunction related to glucometabolic dysregulation may also result from enhanced expression of several endothelial miRNAs (short, single-stranded, non-coding RNA molecules) which mediate gene-regulatory mechanisms in angiogenesis, vascular repair, and inflammation. Obesity has been associated with enhanced expression of miR-24, miR-155, miR-15b, miR-16, miR-221/222, and miR-765, which mediate endothelial dysfunction via direct inhibition of eNOS translation [109,110].

Furthermore, gut microbiota and their metabolites have been suggested to play an important role in vascular homeostasis through different mechanisms, notably by influencing endothelial NO production and bioavailability and the expression of immunoinflammatory mediators [111,112,113,114]. It was shown that gut microbiota can impair endothelium-dependent vasorelaxation by remotely downregulating Sirtuin1 (Sirt 1) and stimulating the expression of vascular miRNA-204, while broad-spectrum antibiotic administration was shown to reverse high-fat diet induced endothelial dysfunction mediated via the microRNA–Sirt 1 nexus [115]. A study of obese children and adolescents noted a significant positive association between endothelial dysfunction markers such as ICAM-1 and VCAM-1 and changes in gut microbiota [116]. Similarly, a cross-sectional study of aging overweight and obese individuals found that independently of BMI, gut microbiota phenotypes correlated positively with vascular endothelial dysfunction as assessed by reactive hyperaemia index [117].

### 3.3. Endothelial and Perivascular Adipose Tissue Inflammatory Mediators

The endothelium and perivascular adipose tissue both secrete vasoactive substances and share important common pathways in the regulation of vascular function (NO, prostaglandins, K^+^ channels, hydrogen peroxide, and hydrogen sulphide) [33]. However, while the role of the endothelium in the regulation of vascular tone has been well established, the mechanisms by which perivascular adipose tissue contributes to microvascular function and dysfunction remains an active area of investigation. Obesity-related chronic inflammatory phenotype is characterized by release of an array of proinflammatory mediators including cytokines (e.g., interleukin-6 (IL-6), interleukin 1β, tumour necrosis factor-α (TNF-α)), and adipokines (e.g., leptins) [118], which promote insulin resistance through alterations in the extracellular matrix, capillary network architecture, and glucose uptake mechanisms. TNF-α regulates insulin-mediated cell signalling, and its increased expression may decrease both insulin-mediated capillary recruitment and glucose uptake in the skeletal muscle by activating the intracellular c-Jun N-terminal kinase (JNK), which attenuates the PI3K pathway and promotes vasoconstriction by activating endothelial ERK-1/2 phosphorylation [119]. Both TNF-α and IL-6 derived from peripheral vascular tissue can stimulate ROS production via activation of NAD(P)H oxidase. Additionally, both inflammatory cytokines can also enhance ROS generation by activating nuclear transcription factor-kappa B (NF-κB) and xanthine oxidase, respectively. It has been further suggested that TNF-α, IL-6, and other inflammatory mediators reduce the production of adiponectin [120,121], an anti-inflammatory adipokine which promotes insulin-mediated vasodilatation through increased eNOS phosphorylation [122]. In concert, these pro-inflammatory mechanisms result in the activation of macrophages, migration, and proliferation of VSMCs, induction of endothelial adhesion molecules such as intercellular adhesion molecule-1 (ICAM1), VCAM-1 and E-selectin, and increased synthesis of endothelin [16,17,123,124]. This suggests that perivascular adipose tissue is an important regulator of vascular homeostasis, and that induction of inflammation represents a pivotal mechanism by which pathological perivascular adipose tissue promotes deleterious effect on the microvasculature [125].

### 3.4. Dysregulation of Redox Homeostasis

The production of ROS in the mitochondria plays a key role in regulating the cellular redox status. Superoxide is the proximal mitochondrial ROS and rapidly undergoes dismutation to yield hydrogen peroxide, which modulates retrograde redox signalling from the organelle to the cytosol and nucleus. Overproduction of ROS in the mitochondria (superoxide and hydrogen peroxide) induces oxidative damage to mitochondrial proteins, membranes, and DNA, consequently impairing mitochondrial ATP synthesis as well as mitochondrial pathways for fatty acid, urea, and amino acid metabolism [126]. Impaired mitochondrial oxidative phosphorylation tilts the cellular metabolism towards greater reliance on glycolytic ATP production with consequent lactic acid accumulation. It has been suggested that endothelial dysfunction and vascular insulin resistance may result from the impairment of cellular adaptive mechanisms against mitochondrial dysfunction and oxidative stress such as the redox-sensitive transcription factor nuclear factor E2-related factor 2 (Nrf2) and the antioxidant response element (ARE), which modulate cellular antioxidant activity [127,128]. Insulin resistance and persistent hyperglycaemia further exacerbate redox dysregulation through a positive feedback loop [129].

The balance between the vaso-protective NO and the vaso-deleterious ROS is disrupted in the setting of hyperglycaemia and insulin resistance [130]. Hyperglycaemia alters the endothelial redox environment by inducing increased ROS generation via several mechanisms, including PKC-dependent activation of vascular NAD(P)H oxidase [62,131]. Skeletal muscles express three isoforms of NAD(P)H oxidases (NOX1, NOX2, and NOX4), which are critically important in the modulation of redox homeostasis [132]. NOX2 generates most of the skeletal muscle ROS during contractions and is involved in insulin signalling and glucose transport [133,134]. In the setting of hyperglycaemia and hyperinsulinaemia, endothelial NOX2 activation promotes vasoconstriction by altering the balance between MAPK-dependent vasoconstriction and PI3K/Akt-dependent vasodilation [133].

In addition to uncoupling eNOS and impairing endothelium-dependent vasodilation, excess ROS derived from NOX1 and NOX2 in the setting of hyperglycaemia also impairs NO production and bioavailability by increasing the production of superoxide anion, which reacts with NO to form peroxynitrite, which in turn oxidises the eNOS cofactor BH_4_ [30]. The superoxide anion further enhances ROS generation via increased formation of glucose-derived advanced glycation end products (AGEs) and activation of the AGE receptor on vascular cells [135]. The build-up of AGEs is pathogenically important in the development of arteriosclerosis. Obesity and insulin resistance also decrease NO production via different mechanisms, including blunting of skeletal muscle eNOS expression and activity, consequently impairing the NO-driven endothelium-dependent vasoreactivity [62]. On the other hand, recent studies have suggested that NO can mediate vasoconstriction rather than vasorelaxation in certain conditions, notably hypoxia, via activation of soluble guanylyl cyclase and consequent production of cyclic inosine monophosphate (cIMP) rather than cGMP [30,136].

Much of the highlighted mechanisms have been derived from preclinical studies, and it therefore remains unclear if the findings can be translated to humans. Other areas requiring clarification in this regard include the relative contribution of hyperglycaemia vs. hyperinsulinaemia in the induction of NOX-derived superoxide production, and the interactions and coordination between the different NOX isoforms and between NOX family and other sources of pathological ROS generation [137].

### 3.5. The Role of Extracellular Matrix Remodelling

The extracellular matrix (ECM) is an important structure in the microvascular environment composed of proteins and proteoglycans. Alterations in this dynamic structure as seen in an inflammatory milieu may mediate skeletal muscle insulin resistance by causing capillary regression and endothelial dysfunction [47]. The chronic inflammatory phenotype seen in obesity and DM type 2 induces compositional changes in the ECM, including increased expression of ECM proteins such as collagen, and glycosaminoglycans such as hyaluronan, which are a major constituent of the capillary luminal endothelial cell glycocalyx [138,139]. The expansion and remodelling of the ECM is associated with capillary rarefaction and insulin resistance. ECM collagen level is inversely related to muscle capillarisation and insulin sensitivity [138,140]. Similarly, decreasing hyaluronan expression using PEGylated hyaluronidase or antibodies against CD44, which is the main hyaluronan cell surface receptor, is associated with improved insulin action [139,141,142].

## 4. Crossroads of Microvascular Pathophysiology and Pharmacology: Clinical Perspectives in Obesity Treatment

### 4.1. Current Paradigm

While lifestyle adjustments like increased physical activity and dietary modification and vigilance remain the fundamental treatment modalities for obesity, such conservative approaches are often insufficient, and adjunctive pharmacological or surgical treatment is usually indicated to realise target clinical outcomes [7,143,144]. Remarkably, conservative obesity treatment measures such as physical activity and healthy dietary habits were even further negatively impacted by the series of lockdown measures instituted to limit the spread of the novel coronavirus [145,146], further highlighting the practical need for supportive medical therapy for obesity and related complications. Unfortunately, although several drugs have been approved for the treatment of obesity over the past few decades, most have been withdrawn due to safety concerns, and only a very limited number are currently available for clinical use [147,148]. Even more far-fetched are treatments rationally designed to counteract the pathways and mechanisms of the chronic effects of obesity. Accordingly, an enhanced understanding of the multiple pathophysiological pathways in obesity will be critical in developing or adapting targeted therapies for obesity-related complications.

Current medications approved by the United States Food and Drug Administration (US-FDA) for the treatment of chronic obesity include orlistat (lipase inhibitor, decreases lipid absorption), phentermine/topiramate (norepinephrine/GABA agonist and glutamate antagonist, which suppress appetite), naltrexone/bupropion (opioid receptor antagonist/dopamine agonist and norepinephrine reuptake inhibitor, which increase satiety and suppress appetite), and liraglutide (glucagon-like peptide-1 (GLP-1) agonist, which promotes slow gastric emptying and satiety) [149,150]. However, phentermine/topiramate is currently not approved by the European Medicines Agency (EMA). In February 2020, the US-FDA ordered the withdrawal of lorcaserin, which until then was one of the most frequently prescribed weight-loss drugs since its approval in 2012, following evidence of increased cancer risks by safety clinical trials [149,151]. Other promising anti-obesity drugs that were withdrawn from the market due to life-threatening adverse effects include aminorex, fenfluramine, dexfenfluramine, phenylpropanolamine, rimonabant, and sibutramine (respectively associated with pulmonary hypertension, cardiac valvopathy, valvopathy, stroke, suicidal ideation and behaviour, and myocardial infarction and stroke) [152]. Although the development and maintenance of obesity and its sequelae are mediated by both central and peripheral mechanisms, most of the currently available pharmacological agents for treatment of obesity act primarily on pathways in the central nervous system, and thus expectedly show a wider potential adverse effect profile in both short- and long-term use [148,149]. Furthermore, the recruitment of alternate and counter-regulatory pathways significantly reduces the long-term efficacy of most of the anti-obesity monotherapies [153].

### 4.2. Targeting Microvascular Inflammatory Phenotype and Endothelial Dysfunction as a Therapeutic Strategy for Insulin Dysfunction in Obesity

Given the limitations of centrally acting anti-obesity medications, specific or adapted therapies targeting the peripheral mechanisms of obesity-related complications seems attractive in terms of risk/benefit balance and the possibilities of tailoring therapy towards the specific downstream metabolic effects of chronic obesity. The mechanisms of the intimate reciprocal relationship between microvascular and metabolic pathophysiology in obesity provides a promising window for pharmacotherapeutic exploitation.

#### 4.2.1. Current Anti-Obesity Drugs

A few studies have evaluated the effects of some of the currently available anti-obesity drugs on inflammatory markers in obese and insulin-resistant patients. The modulation of visceral and vascular inflammatory phenotypes may bear indirect therapeutic relevance to microvascular dysfunction. It was shown that treatment with orlistat for at least 6 months was associated with reduction in serum IL-6, TNFα and high-sensitivity C-reactive protein (hsCRP) levels [154,155]. This anti-inflammatory effect appears to strongly correlate with the degree of weight loss over the treatment duration. Data on the anti-inflammatory properties of naltrexone/bupropion are inconsistent with different trials reporting reductions in hsCRP or no significant change [156]. In the CONQUER trial, phentermine/topiramate was associated with decreased hsCRP and increased adiponectin levels [157].

A randomized, double-blind, placebo-controlled trial in DM type 2 patients with persistent albuminuria showed that liraglutide treatment for 12 weeks reduced TNFα and mid-regional pro-adrenomedullin levels [158]. Liraglutide was also shown to mediate modulatory effects on inflammatory gene expression in peripheral blood mononuclear cells [159]. Using [^64^Cu] DOTATATE, a novel high-resolution PET tracer, it was recently suggested in a randomized placebo-controlled study that liraglutide treatment reduced vascular inflammation, which is a probable mechanistic explanation of the clinically observed cardiovascular protective effect of GLP-1 receptor agonists [160]. However, this study was limited by a small sample size and the lack of statistical significance in the observed effect. In obese patients with DM type 2, Liraglutide treatment was associated with inhibition of NF-κB pathways and up-regulation of Sirt1 expression, and decreased levels of inflammatory markers such as TNFα and ceruloplasmin [161]. Conversely, compared to the placebo group, 26-week liraglutide treatment in a low- to moderate-risk population DM type 2 patients did not change vascular inflammation as assessed by [^18^F]-fluorodeoxyglucose PET-CT, although an explorative analysis indicated a possible effect in patients with pre-existing background of cardiovascular disease [162]. Similarly, a 12-week treatment with liraglutide yielded no effect on capillary perfusion or vasomotion in diabetic patients, suggesting that the glycaemic effects of GLP-1-based therapies may be independent of microvascular responses [163]. Further investigations are therefore warranted to clarify the role of GLP-1 receptor agonists in microvascular response.

#### 4.2.2. Anti-Hyperglycaemic and Other Agents

Several other antihyperglycemic agents, notably metformin, dipeptidyl-peptidase (DPP)-4 inhibitors (e.g., vildagliptin, linagliptin), GLP-1 analogues (e.g., exenatide), and sodium-glucose cotransporter 2 inhibitors (SGLT2i) (e.g., empagliflozin), have also been suggested to confer microvascular protective benefit related to, or independent of, glycaemic control mechanisms. Metformin is the first-line drug for treating patients with DM type 2 and is increasingly also used for clinical management of other insulin-resistant states such as prediabetes and polycystic ovarian disease [164] on account of known cardiovascular benefits and pleiotropic effects. A growing body of evidence suggests that metformin improves vascular endothelial dysfunction via AMPK dependent and independent mechanisms, including downregulation of NF-κB and upregulation of PI3K-Akt-eNOS, Sirt1, forkhead box O1 (FOXO1), and krüppel-like factors (KLF) 2 and 4 [29]. Compared to control, obese diabetic patients treated with metformin expressed lower levels of inflammatory markers such as hsCRP, TNF-α, and Toll-like receptors 2/4 [165]. In a recent study on obese newly diagnosed drug-naïve DM type 2 women, metformin treatment for 30 days was associated with increased nutritive microvascular reactivity and functional capillary density during post-occlusive reactive hyperaemia [166].

Metformin and vildagliptin have been suggested to exert microvascular effects via distinct but potentially complementary mechanisms. Following ingestion of a lipid-rich meal, metformin, but not vildagliptin, was shown to increase functional capillary recruitment in obese patients with DM type 2 [167]. Vildagliptin on the other hand increased endothelial-dependent and -independent vasodilatations at the arteriolar level, following 30 days of treatment in obese diabetic women [166]. Conversely, a multicentre, prospective, randomized, parallel-group comparison of double-dose metformin (1–1.5 g/d) vs. low-dose metformin (0.5–0.75 g/d) plus add-on vildagliptin in DM type 2 patients found that combination therapy of vildagliptin and metformin had no effect on endothelial function as assessed by flow-mediated dilation before and after 12 weeks of treatment, although favourable effects on adipokine levels were noted [168]. Furthermore, Petrie et al. also noted that regardless of a wider role in cardiovascular risk management, metformin treatment in patients with long-standing DM type 1 had no effect on endothelial function as assessed by reactive hyperaemia index, or on retinopathy [169]. While linagliptin showed no effect on macrovascular function, it was significantly associated with improved fasting-state microvascular function in DM type 2 patients [170]. An ongoing multinational, randomised, partially double-blind, placebo-controlled clinical trial on the effect of lifestyle and pharmacological interventions on early prevention of hyperglycaemia-related microvascular complications will hopefully shed new light on the effects of metformin and linagliptin on microvascular function in people with prediabetes [171].

Three-month therapy with the GLP-1 receptor agonist exenatide showed a similar effect to metformin on microvascular endothelial function, inflammatory phenotype, and redox homeostasis, as assessed by reactive hyperaemic index, C-reactive protein (CRP), circulating oxidized low-density lipoprotein, and VCAM-1 [172]. However, in obese patients with insulin resistance, acute treatment with exenatide following a high-fat meal was associated with blunted postprandial vasodilatory response [173]. On the other hand, SGLT2 inhibitors such as empagliflozin, canagliflozin, and dapagliflozin, which are clinically remarkable for their favourable cardiovascular and renal profile in diabetic patients [174], have additionally been suggested to have benefits in obesity. In high-fat-diet-induced obese C57BL/6J mice, it was shown that empagliflozin significantly reduced whole body weight and fat, improved metabolic function, and ameliorated obesity-related myocardial hypertrophy/fibrosis and dysfunction [175]. These effects were mediated via upregulation of Sestrin2-mediated increase in AMPK and eNOS phosphorylation and inhibition of Akt and mTOR phosphorylation. Sestrin2 is a stress-inducible protein that regulates AMPK-mTOR signalling and redox homeostasis. However, in patients with DM type 2 and cardiovascular morbidity, empagliflozin treatment for 24 weeks had no effect on peripheral endothelial function, suggesting that its cardiovascular benefits may be attributed to other mechanisms rather than improvement in endothelial function [176].

Targeting the mammalian Sirt1, which reciprocally activates AMPK to inhibit lipid accumulation and stimulate fatty acid oxidation, has also been proposed as a therapeutic option in obesity. L-leucine and metformin are a known allosteric activator and a synergistic coactivator of Sirt1, respectively, while sildenafil is a phosphodiesterase-5 inhibitor and vasodilator which indirectly stimulates Sirt1 by increasing NO bioavailability. The Leucine–Metformin–Sildenafil fixed-dose combination is a pharmacologic attempt to synergistically exploit these mechanisms, and recent randomized control trials noted significant weight reduction in obese non-diabetic patients treated for 16 and 24 weeks [177,178].

It has also been shown that alpha adrenergic blockers (e.g., prazosin) can mediate capillary growth in human skeletal muscles via increased shear stress [179,180]. While this angiogenic effect may beneficially counteract structural capillary regression and its metabolic sequelae in obesity, the pharmacodynamic mechanisms exploited here are not directly related to the microvascular metabolic mechanisms described in the pathogenesis of obesity-related insulin resistance. Furthermore, given the contribution of the renin–angiotensin–aldosterone system to the pathogenesis of endothelial dysfunction, angiotensin-converting enzyme inhibitors (ACEi, e.g., lisinopril) and angiotensin receptor blockers (ARBs, e.g., losartan) have been shown to exert microvascular protective and insulin resistance counteractive effects beyond their basic antihypertensive actions. Besides improving endothelial function and redox homeostasis, the peripheral vasodilatory actions of ACE inhibitors and ARBs contribute to enhanced skeletal muscle blood flow. A meta-analysis of 12 randomized controlled clinical trials of ACEi or ARBs found that both antihypertensive medication types decreased the incidence of new-onset diabetes by 27% and 23%, respectively, highlighting a significant clinical benefit in patients with prediabetic conditions such as obesity and metabolic syndrome [181].

#### 4.2.3. Experimental Phytochemicals and Dietary Interventions

Several medicinal herbs have been suggested to have therapeutic benefit in vascular endothelial dysfunction, notably via anti-inflammatory, anti-oxidative, and anti-apoptotic effects. For example, traditional Chinese medicinal herbs like Danshen (Salvia miltiorrhiza), Shanchi (Panax notoginseng), Shanzai (Hawthorn), and Heshouwu (Polygonum multiflorum Thunb) were shown to decrease apoptosis and inhibit adhesion molecule expression in human umbilical vein endothelial cells [182]. Similarly, Naoxintong, a compound herbal mixture containing Radix Astragali, Angelicae sinensis, Paeoniae radix rubra, and Ligusticum wallichii, was shown to improve the protective effect of high-density lipoprotein on endothelial function in DM type 2 patients [183]. Hydroalcoholic extract of Teucrium polium, a traditional antidiabetic medicinal herb, improved endothelial dysfunction by regulating vasoreactivity and eNOS and VCAM-1 genes’ expression in streptozocin-induced diabetic rats [184]. However, the specific chemical compounds responsible for the putative pharmacologic effect of the herbal extracts, and their toxicological properties, are yet to be identified and characterized.

Other bioactive compounds have also been suggested to exert microvascular protective actions in obesity via favourable effects on various microvascular dysfunction pathophysiological mechanisms described above. A typical example is resveratrol, a naturally occurring polyphenolic phytoalexin found in red wine that modulates endothelial function by targeting AMPK, eNOS, nuclear factor-erythroid-derived 2-related factor-2 (Nrf2), KLF2, and NF-κB [185]. In addition, polyphenol compounds such as chlorogenic acid, piceatannol, taxifolin, quercetin, fisetin, kaempferol, and caffeic acid have been shown to inhibit arginase activity and enhance endothelial function by increasing NO levels and decreasing ROS generation [186]. Other naturally occurring bioactive compounds with suggested beneficial effects in microvascular dysfunction include garlic, cinnamon, olive, extra virgin olive oil, ginger, cocoa (modulation of endothelial function), hydroxytyrosol, oleocanthal, and quercetin (modulation of inflammation and oxidative stress) [187]. Furthermore, given the increasing recognition of the role of gut microbiota in pathogenesis of vascular endothelial dysfunction, several interventions targeting gut dysbiosis have been suggested, including high-fibre diet, zinc supplementation, use of pre- or probiotics and faecal microbiota transplantation [188].

## 5. Conclusions

In this review, we discussed the current evidence on the relationship between skeletal muscle microvascular dysfunction and insulin resistance in obesity. Several reciprocal and interconnected pathways were shown to intimately link microvascular physiology and metabolic functions, with the delicate balance in these pathways disrupted in obesity. Several intertwined mechanisms, including endothelial cell dysfunction from various factors, induction of immuno-inflammatory cascades in endothelial cells and perivascular adipocytes, dysregulation of redox hemostasias and extracellular matrix remodelling, are thought to mediate obesity-related structural and functional alterations in skeletal muscle microcirculation and contribute to insulin dysfunction and glucose dysregulation. We further reviewed the therapeutic implications thereof by correlating the explored peripheral pathophysiological mechanisms with clinical and pharmacodynamic data on both currently approved and adapted medications for treatment of obesity and its complications.

While several preclinical studies have suggested a close link between microvascular and metabolic dysfunction in obesity, overall, the paucity of clearcut prospective evidence for many of the suggested mechanisms means that direct causal effect awaits conclusive proof. Similarly, while many of the examined anti-obesity medications appear to make pathophysiological sense, remarkable inconsistencies in the clinical data question suggested effects and benefits. Nevertheless, adapting existing or developing novel therapies targeting peripheral mechanisms such as the pathophysiological interface between skeletal muscle microvascular and metabolic function in obesity still represents a rational perspective in obesity pharmacotherapy requiring further exploration.

## Data Availability

Not applicable.

## Abbreviations

AMPK	5’-Adenosine Monophosphate-activated Protein Kinase
Akt	Protein Kinase B
AGEs	Advanced Glycation End Products
Ang	Angiotensin II
ARBs	Angiotensin Receptor Blockers
ARE	Antioxidant response element
BMI	Body Mass Index
CRP	C-Reactive Protein
DM	Diabetes Mellitus
DPP4i	Dipeptidyl-Peptidase-4 inhibitors
ERK	Extracellular-signal-regulated Kinase
eNOS	Endothelial Nitric Oxide Synthase
ET-1	Endothelin 1
FOXO1	Forkhead Box O1
GABA	Gamma-Aminobutyric acid
GLP-1 RA	Glucagon-like Peptide-1 Receptor Agonists
GLUT-4	Glucose Transporter 4
hCRP	High-Sensitivity CRP
HMGB 1	High Mobility Group Box chromosomal protein 1
IRS	Insulin Receptor Substrate
ICAM1	Intercellular Adhesion Molecule 1
IL-1β	Interleukin-1Beta
IL-6	Interleukin-6
JNK	C-Jun N-terminal Kinase
KLF2	Krüppel-Like Factor 2
KLF4	Krüppel-Like Factor 4
mTOR	Mammalian Target of Rapamycin
MAPK	Mitogen-Activated Protein Kinase
MCP1	Monocyte Chemoattractant Protein 1
miRNAs	MicroRNAs
NADPH	Nicotinamide Adenine Dinucleotide Phosphate
NF-κB	Nuclear Factor-KappaB
NO	Nitric Oxide
NOX	NADPH Oxidase
Nrf2	E2-related factor 2
PDE-5i	Phosphodiesterase-5 inhibitors
PET-CT	Positron emission tomography—computed tomography
PI3K	Phosphatidylinositol 3 Kinase
PKC	Protein Kinase C
ROS	Reactive Oxygen Species
RAGE	Receptor for AGEs
SGLT2i	Sodium Glucose Cotransporter 2 inhibitors
SIRT1	Sirtuin 1
TXA2	Thromboxane A2
TNF-α	Tumour Necrosis Factor-A
VCAM1	Vascular Cell Adhesion Molecule 1
VEGF	Vascular Endothelial Growth Factor
VSMCs	Vascular Smooth Muscle Cells
